# Scrolling Through the COVID-19 Pandemic: Exploring the Perceived Effects of Increased Social Media Use on the Mental Health of Undergraduate University Students

**DOI:** 10.1177/20563051231177970

**Published:** 2023-06-13

**Authors:** Dario Giancola, Robb Travers, Simon Coulombe

**Affiliations:** 1 Wilfrid Laurier University, Canada; 2 Université Laval, Canada

**Keywords:** COVID-19, mental health, Ontario, social media, university students

## Abstract

Social media has become increasingly integrated into the lives of students for the past decade; however, the public health restrictions associated with the COVID-19 pandemic have led to a sharp increase in social media use in a short period of time. The purpose of this study was to investigate the effects of social media use on university students during the COVID-19 pandemic. Fifteen students from a mid-sized Canadian city were interviewed to share their experiences with social media during the COVID-19 pandemic. Purposive sampling was conducted to gather a diverse sample of participants, including individuals of various ages, gender and sexual identities, and ethnicities. Thematic analysis on the 15 interviews was completed using NVivo (version 12). Participants experienced both advantages and disadvantages associated with social media use. Ease of communication and stress relief were acknowledged as the strongest benefits. Social comparison, loneliness, development of bad habits, and lack of focus were cited as major disadvantages to social media use during the pandemic. Cost–benefit analysis of social media was common, and participants expressed the importance of using social media with moderation, balance, and awareness. Our study indicates that the focus on health with respect to the pandemic should not be solely based on physical health, rather the potential mental health risks associated with social media use during the pandemic should be recognized and addressed by healthcare providers.

## Background

Physical health has been a substantial focus of healthcare professionals during the pandemic. In addition, research has been conducted to examine the consequences of the pandemic on mental health. Da Silva Junior et al. (2020) state the psychological effects associated with the pandemic can last longer and be more determinantal than the infection itself. The public health measures and social restrictions implemented by various governments worldwide have resulted in adverse mental health outcomes. In Australia, moderate to severe symptoms of depression and generalized anxiety doubled in regions where COVID-19 restrictions were most severe (Fisher et al., 2021). In Ireland, young people were particularly vulnerable to depression, feelings of social isolation, and anxiety during periods of social restriction (O’Sullivan et al., 2021). In addition, the same study found that children with autism spectrum disorders experienced worsening mental health mostly due to changes in routine demonstrating the impact of social restrictions on vulnerable populations of society (O’Sullivan et al., 2021). Even where researchers documented a decrease in mental health emergency department visits and crisis calls throughout the first wave of the pandemic, they acknowledge that data may present differently if collected over a longer period as an accumulation of stress, loneliness, isolation, and other factors may have taken longer to develop (Dainton et al., 2021). With respect to Canadian data, Statistics Canada’s Survey on COVID-19 and Mental Health (SCMH) showed that the proportion of Canadian adults screening positive for major depressive disorder (MDD) increased from 15% in fall 2020 to 19% in spring 2022 (Statistics Canada, 2021). The proportion of Canadian adults screening positive for general anxiety disorder (GAD) also increased from 13% in fall 2020 to 15% in spring 2022 (Statistics Canada, 2021); younger Canadians aged 18 to 24 were vulnerable to adverse mental health outcomes during the COVID-19 pandemic as a higher proportion reported experiencing feelings of loneliness.

Mental illness has been, and continues to be, prevalent in adolescents, which represent a significant proportion of university students. According to the National Institute of Mental Health (2023), those in the 18- to 25-year age group reported the highest prevalence of any mental illness compared to adults in the 26- to 49- year age group and the 50 and older age group. Approximately one in five adolescents suffer from a diagnosable mental disorder demonstrating a heightened risk for young adults (Nesi, 2020). In addition, the incidence rates of depression and suicide have increased significantly within the past decade (Nesi, 2020). Data on suicide in the United States reveal a 56% increase in suicide rate among individuals aged 10 to 24 from 2007 to 2017 (Curtin & Heron, 2019). The consequences of the pandemic present new mechanisms by which the mental health of university students is impacted. Visser and Law-van Wyk (2021) reported that university students in South Africa experienced challenges related to mental and emotional health during the pandemic as a third of the respondents had difficulty coping with psychological challenges presented in the lockdown. Furthermore, emotional difficulties during the lockdown especially impacted students early in their university education compared to more experienced students (Visser & Law-van Wyk, 2021). A study conducted in Georgia found reported serious mental health impacts from the pandemic as 79% of respondents reported experiencing anxiety and 46.7% reported experiencing depression (Nadareishvili et al., 2022). University students who are women seem to be at a higher risk for experiencing negative mental health symptoms compared to men (Nadareishvili et al., 2022; Visser & Law-van Wyk, 2021). According to Nadareishvili et al. (2022), possible protective factors mitigating the negative impacts of the COVID-19 lockdown on mental health include reduced fear of COVID-19, less family conflicts, reduced substance use, and absence of nightmares. A study conducted at Queen’s University and the University of Oxford found that approximately 30% of participants at Queen’s University and 14% of participants at the University of Oxford had activities important in maintaining good mental health disrupted by the pandemic (Appleby et al., 2022). Despite physical and social restrictions, an easily accessible activity is using social media.

Social media is a relatively new phenomenon that has emerged as a product of increased communication capacity within technology. The advent of smart phones has allowed for communication to take forms that are more entertaining than traditional forms of communication such as phone calls or letters. Social media can be regarded as a modern form of communication, allowing users to chat, call, follow particular people or organizations, and express themselves. In Canada alone, 9 in 10 individuals aged 15 to 34 use social media regularly (Schimmele et al., 2021). With respect to students, information and communication technologies (ICTs) have become a foundational component in education and communication for young people (O’Reilly et al., 2018). With the emergence of ICTs, many studies have been published looking into the effects of social media on users and have found both positive and negative outcomes. It is evident that the quality (versus quantity of use) of social media use is a stronger determinant of positive versus negative outcomes (Berryman et al., 2018).

Social media use has been linked to various negative mental health outcomes. Mental health problems including depression and anxiety have been linked to social media use (Barry et al., 2017; Hanprathet et al., 2015; Iqbal et al., 2022; Tsitsika et al., 2014; Yan et al., 2017). Although, it is important to note that the mechanism is multifactorial and these relationships are complex (Keles et al., 2020). With respect to university students, social media use has increased substantially as the use of the internet for education has become essential (Haddad et al., 2021). Elementary and high school students who experience cyberbullying, a common and concerning aspect of online behavior and social media, are more likely to have suicidal ideation, plans, and attempts compared to those who do not experience cyberbullying (Sampasa-Kanyinga et al., 2014). Tsitsika et al. (2014) found that heavier social media use was associated with diminished academic performance. Sleep disruption and deprivation are linked with excessive social media use and negative mental health outcomes (Abi-Jaoude et al., 2020; Alonzo et al., 2021; Schimmele et al., 2021) including anxiety, depression, and psychological distress, and was especially prominent in those aged 16 to 25 (Alonzo et al., 2021). Another potential negative outcome associated with social media use is an increased sense of loneliness which has been demonstrated through multiple studies (Barry et al., 2017; Hunt et al., 2018; Thomas et al., 2020). Hunt et al. (2018) found that a significant decrease in loneliness and depression was observed in undergraduate students that limited their social media use to 30 min per day compared to the control group who did not. Studies completed in North America found that online social comparison can lead to the development of depressive symptoms through various mechanisms (Boers et al., 2019; Feinstein et al., 2013; Nesi et al., 2017).

Despite the negative associations described in the literature, social media’s role in the development of negative mental health outcomes remains a subject of debate and contention. The literature conveys mixed results with respect to mental health outcomes. A recent study of 467 undergraduate students in the United States found that mental health problems including social anxiety, suicidal thoughts, loneliness, and others, could not be predicted by social media use (Berryman et al., 2018). Furthermore, Nesi (2020) posits that social media conjures an environment capable of providing benefits to the user including entertainment, creative expression, and social connection. Social media also offers a unique environment for mental health awareness, promotion, and treatment (Nesi, 2020; O’Reilly et al., 2019). Adolescents and university students spend significant amount of time on social media which makes the platforms an ideal place for mental health promotion campaigns despite the negative implications of social media use (O’Reilly et al., 2019).

### The Current Study—Context

On 11 March 2020, the World Health Organization officially declared COVID-19 a pandemic. The provincial governments of Canada implemented various public health protocols and social restrictions to mitigate the spread of the SARS-CoV-2 virus including the closure of non-essential businesses and public facilities including parks and recreational spaces and playgrounds (Lesser & Nienhuis, 2020). The federal government of Canada also implemented public health measures including restricting international travel and strongly recommending self-isolation for exposed individuals (Lesser & Nienhuis, 2020). Although public health measures varied between provinces, lockdown measures were implemented frequently throughout the pandemic timeline as new waves of variant strains threatened to overwhelm the provincial healthcare systems.

## Research Objectives

The current study seeks to expand on the existing literature through an in-depth, qualitative exploration of how social media has affected university students during the COVID-19 pandemic. The pandemic has provided a new avenue for research as new factors effect social media consumption as a result of the pandemic and the social/public health restrictions implemented to control it.

The purpose of this study was to develop a greater understanding of how social media usage has affected (and continues to) university students during the COVID-19 pandemic. The objectives of this research were (1) to explore why participants were using social media and to understand the nature of their engagements on various platforms; (2) to investigate how participants perceive that social media use may have affected their mental health in both positive and negative ways; and (3) to recommend strategies for the integration of discussion about healthy social media use into various educational forums. In this study, social media was defined as “forms of electronic communication through which users create online communities to share information, ideas, personal messages, and other content” (Merriam-Webster, 2022). This definition captures all traditional forms of media that allow for social networking including Facebook, Instagram, Twitter, and TikTok, but excludes common media streaming platforms such as Netflix, Disney Plus, and Amazon Prime Video. This definition was ideal as most university students are using platforms that are encompassed by it.

## Methods

The study was approved by Wilfrid Laurier University Research Ethics Board (REB #7086). Primary data were collected by the student researcher, Dario Giancola, with supervision from Dr. Robb Travers.

### Participants

This study was completed using a purposive sampling method of students attending a mid-sized Canadian university in a smaller urban-rural region. Eligibility for the study was determined using three criteria: (1) participants must have been enrolled at Wilfrid Laurier University, (2) participants must have been familiar with 3 or more social media platforms, and (3) participants must have observed an increase in their social media use during the pandemic. Fifteen individuals were interviewed.

Criterion 2 required that individuals be familiar with three or more social media platforms to gain various perspectives with respect to different platforms. An individual’s experience on Instagram may be different than their experience on Snapchat. Thus, this criterion was implemented to acquire perspectives across platforms. Furthermore, Criterion 3 was implemented to directly investigate how an increase in social media usage impacted individuals. A decrease in usage should be investigated in a separate study.

### Recruitment

Participants were recruited using promotional emails to student email listservs at Wilfrid Laurier University. The promotional email contained information explaining the nature of the study and a Qualtrics survey. Within the Qualtrics survey, was the informed consent document, which contained important information about the study. Upon providing consent, participants completed a screening questionnaire to collect demographic and contact information. Sociodemographic data collected included age, gender, ethnoracial identity, and sexual orientation and gender identity. Purposive sampling was completed using these sociodemographic details to ensure diversity within the study population. In addition, the screening questionnaire queried participants’ social media usage. Those who met the eligibility criteria were contacted for an interview and provided additional information concerning the study.

### Interviews

Semi-structured interviews were conducted with participants during February 2022; the average interview time was 37 minutes with a range of between 26 and 53 minutes. The interviews were conducted by the student researcher; the participants were offered an alternative interviewer if they felt uncomfortable being interviewed by the student researcher. This option was implemented as there was potential for the student researcher to be a direct peer of the participant. An interview guide that included questions about social media use and mental health was used (see Appendix). Sample questions included: (1) what are some of the disadvantages of using social media during the pandemic? (2) What are some of the benefits of using social media during the pandemic? (3) What are the reasons for your social media use? The interviews were conducted over Zoom with various privacy protocols in place and participants received a $10 gift card to a popular coffee chain as compensation for their time.

## Data Analysis

Interviews were transcribed by Transcript Heroes, a transcription organization located in Toronto, Ontario, Canada. Confidentiality agreements were signed on behalf of the student researcher and Transcript Heroes to preserve and maintain confidentiality throughout the transcription process. Coding was completed by the student researcher using NVivo (version 12), a qualitative data analysis software developed by QSR International. Upon the development of the codes, major themes were extracted, and a thematic framework was created using codes from all 15 interviews.

## Results

### Sample Characteristics

Fifteen individual interviews were conducted with a diverse sample of university students. A majority (73.3%) of participants identified as female. Participant’s age ranged from 17-22 with 60% between 19-20. Most participants included in the analysis were Asian, South Asian, or Caucasian, each representing approximately 26.7% of the sample. Other ethnoracial identities included Latin American and Middle Eastern/North African which represented about 20% of the sample combined. Of those reporting sexual identity, 60% identified as heterosexual, with one participant identifying as bisexual, and one participant identifying as homosexual. Table 1 provides the complete set of sample characteristics.

**Table 1.** Sample Characteristics.

Characteristic	Number of participants (*n* = 15)	Percentage of participants
Gender identity		
Male	3	20.0
Female	11	73.3
Non-binary	1	6.7
Age		
17–18	3	20.0
19–20	9	60.0
21–22	3	20.0
Ethnoracial identity		
Middle Eastern/North African	2	13.3
Asian	4	26.7
Caucasian	4	26.7
Latin American	1	6.7
South Asian	4	26.7
Sexual identity		
Heterosexual	9	60.0
Homosexual	1	6.7
Bisexual	1	6.7
Prefer not to say	1	6.7
Unknown/missing	3	20.0

## Qualitative Data

**Figure 1.**
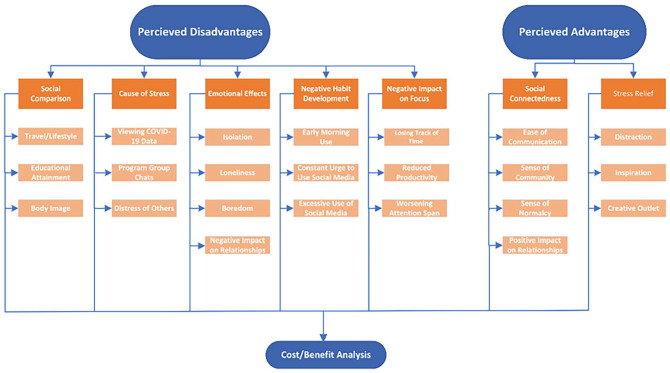
Perceived advantages and disadvantages of using social media during the COVID-19 pandemic. Microsoft Visio was used to create this diagram.

Figure 1 depicts the major themes extracted from the interviews that were associated with social media use during the COVID-19 pandemic. All major themes were categorized based on their advantageous and disadvantageous impacts according to the participants of the study. As seen in Figure 1, the disadvantageous impacts of using social media were more common compared to the advantageous impacts. Social media use during the pandemic negatively impacted the mental health of individuals through mechanisms of social comparison, stress development, symptoms of isolation/loneliness, poor habit development, and difficulty focusing on tasks. Furthermore, sub-themes extracted from these major themes included lack of attention span, development of a constant urge to check social media accounts, and one comparing their lifestyle to others seen on social media. Perceived advantages of using social media included feelings of social connectedness and stress relief. Social media seemed to provide individuals with a sense of normalcy as they were able to interact with others online.

### Perceived Disadvantages of Social Media Use During the Pandemic

#### Social Comparison

Social comparison was a frequent and significant issue discussed in the interview process. Traditionally, body comparison was the primary form of social comparison on social media. However, some participants also discussed lifestyle comparison.

I feel like ever since COVID has hit the comparisons strive to more what people are doing with their lives and what they’re successes are and what their failures are. Which I think is equally, if not more destructive, then comparing body image. (Female, 21–22)

Social media platforms allowed participants to observe the lifestyles of others, many of which were not under the same public health restrictions as the Ontario participants. Most participants agreed that viewing others on social media traveling and engaging in social events was difficult and often resulted in feelings of loneliness or isolation. Many participants reported feeling as though they were bypassing important aspects of their youth due to the public health measures in place.

So, it kind of created FOMO (fear of missing out) in a way, because . . . I was here not being able to do anything, not being able to go out to restaurants, not being able to see anybody, not being able to gather more than 10 people. And they were at, live in Miami, which is a massive club, partying and doing all the things that I used to love to do before the pandemic when I was able to. (Female, 19–20)

Participants reported that social comparison occurred not only through observation of friends, family, and peers on social media but also through observation of celebrities and influencers who have a large social media presence.

I think the major thing is. . . influencers, they show off. . .their wealth, their success, their happiness in relation to that financial or monetary success. And so it’s really easy to watch them, enjoy them, and you feel like you’re part of that but. . .your real life isn’t like that and you could be. . .living in the worst place possible. (Male, 19–20)

I don’t know if the correct terms are jealousy or like envy but you might feel like. . .your life isn’t as grand as it could be or. . .you made some mistakes in the past and now you see. . .your friends doing better than you are, or things like that. And that can weigh you down as well. (Male, 19–20)

Another common aspect of social comparison discussed by the participants was the comparison of educational attainment and the future ambitions of others. It is common for students to post about their successes at a university or educational institution, as well as post about their acceptances to other universities. For example, upon completing their undergraduate degree, it is common for an individual to post about their acceptance into a graduate program.

when I see someone in the same field that I want to go into. . . get in, obviously I do feel happy for them, but it stresses me out because I’m like, “What am I doing? I’m scrolling through TikTok.” It always come to mind that I’m scrolling through TikTok and watching their videos. (Female, 21–22)

Participants discussed how body comparison through social media negatively impacted their well-being during the pandemic. The public health measures in Ontario denied gyms and exercise facilities from opening, preventing many from completing their usual exercise routine. Although body comparison has always been a major aspect of social media, the restrictions preventing participants from exercising seemed to exacerbate the effects as a sense of helplessness was felt.

if you see someone with a. . .nice body. . .you can be like OK; at least I can go to the gym and like try to. . .do that. But when you’re at. . .a stay-at-home order and you see people. . .living their best lives, and. . .[have] a perfect body, perfect hair. . .and you can’t go anywhere. . .you feel like you can’t do anything. (Female, 21–22)

#### Cause of Stress

Participants discussed how social media can both cause and relieve stress. About half of the individuals reported social media as a stressor, while the other half believed that social media was a stress reliever. Despite the difference, participants reported different mechanisms through which social media can cause stress.

Viewing COVID-19-related data was a stressor early in the pandemic but was less impactful as time passed. Participants reported that the initial fear associated with the pandemic, which could be observed through social media posts, did cause stress. However, as participants became accustomed to an altered lifestyle and learned more about the virus and local/global situation, COVID-19 data became less of a stressor.

I feel like especially on Twitter like seeing all the live reports of cases in Ontario or in Canada. I see numbers. It definitely does scare me because I know that our healthcare system is basically being stretched to the absolute limit and there is very little we can do. (Non-binary, 19–20)

I feel like that since we’ve been in lockdown for so long, and like I don’t want to say we got accustomed to like seeing the case count, but like we kind of have. The worry has reduced a bit, and plus. . .I feel more comfortable. . .having three vaccines and then already having COVID before. (Female, 21–22)

Participants also reported experiencing stress from school-related activity through social media. Group chats, where individuals would discuss classes, grades, opportunities, and more, resulted in stress.

there’s obviously going to be people smarter than you, and who excel at different things than you. Seeing it. . .plastered on every group chat, people knowing the answers to things, people. . .doing co-op opportunities, and all this. It definitely makes it seem like there’s a lot of competition, which can be stressful. (Female, 19–20)

A small number of participants expressed how observing others in distress through social media during the pandemic impacted them negatively. One discussed how seeing others in distress went beyond the COVID-19 pandemic and into the realm of socio-political tensions due to the death of George Floyd in 2020.

the biggest point of stress is seeing how the pandemic is affecting other people or just seeing how they’re being affected. (Female, 19–20)

especially during the Black Lives Matter protests seeing. . .if you see people that. . .you can relate to, like suffering and stuff like that you in turn feel like more stressed and anxious. (Female, 21–22)

#### Emotional Effects

A majority of participants reported feelings of loneliness associated with the initial lockdown period. The transition of class time to an online setting required students to stay at home for longer periods of the day with minimal socializing. For students living away from their family, many moved back during the lockdown periods and reported their move as being mentally difficult. Social media was beneficial to some in relieving feelings of loneliness as it provided a communication outlet.

I was going through a really tough bit during the wintertime in the spring, so I had quite a few talks with my friends and they were just supporting me. And I don’t think I would have had quite as many if it wasn’t through social media. (Male, 17–18)

In contrast, social media use was disadvantageous in relieving feelings of loneliness as some individuals would observe social events through social media platforms which elevated their feelings of loneliness as they were unable to experience those same events due to public health restrictions.

seeing everyone on the internet like hanging out and going out and stuff like that kind of gave me a feeling of—I would say yeah, isolation and loneliness, because it was like everyone else is going on trips and they’re going out, and they’re travelling, and they’re going to restaurants, and they’re going to hotels, and they’re all having a great time and I kind of wasn’t. (Female, 21–22)

Most participants agreed that the main contributor to their increase in social media usage was boredom caused by public health restrictions. Social media provided a source of entertainment that was quick, accessible, and constantly present. The public health measures implemented in Ontario prevented social interaction in-person causing many students to seek entertainment and social engagement online.

I found myself. . .extremely bored. There was nobody to talk to and you couldn’t talk to anybody unless you went online. . .especially just the ease of being able to talk to people online, combined with just how you can’t talk with anybody any other way. (Male, 17–18)

Participants reported difficulty in making new friendships as well as maintaining already established friendships. The lack of in-person interactions, such as attending classes, made it significantly more difficult for individuals to form meaningful connections with new people. Participants outlined the utility of in-person events on the university campus for meeting new people and expanding their social networks. Online interactions, through platforms such as Zoom, were not useful in creating long-term friendships. Furthermore, some participants reported difficulty in maintaining relationships due to a lack of in-person interaction. In contrast, some participants found it easier to maintain already solidified relationships.

I would definitely say my communication with others kind of dropped off more than it would have if we were in person, just because I didn’t care to like sit behind my computer and talk to somebody. . .I do that all day, I don’t need to do that with my friends in my spare time. (Female, 19–20)

#### Social Media Use as Habitual

A common behavior reported by most participants was using their cell phones upon waking up or early in their day. Scrolling through various social media platforms upon waking up became a behavior. The amount of time they spent on the platforms upon waking up varied across participants and some stated negative outcomes associated with the behavior including observing posts that caused concern and impacted the rest of their day.

I always check social media probably first thing the morning. . .The whole inflammatory situation. . .you wake up you check your phone, you see. . .posts about, I don’t know whether it’s COVID cases rising. . .political issues or something. . .and those issues. . .get you inflamed for the morning and for the rest of the day. . .you only think about that. I think that’s a very negative impact. (Male, 19–20)

Participants also reported using social media consistently throughout the day and feeling a sense of urgency to check their social media platforms often. They acknowledged that the tendency was habitual, stating that often there was no reason to check their social media platforms; they simply felt a strong desire to open the app regardless of the situation.

Even during class, you know like I find myself leaning towards wanting to go on my phone, like you just feel like you have tendencies to just—even if you know there is nothing happening, I just feel like I need to just unlock my phone and just see the app. Even if it’s for five seconds I just have—like it just scratches an itch in my brain. (Non-binary, 19–20)

#### Inability to Focus

Participants spoke of the time investment involved in using social media. Many reported using social media for extended periods of time, while lacking awareness of their usage. Participants acknowledged the ease of which social media passes time and how hours could pass while scrolling without them noticing.

I noticed from when I would go on it, I would be on it for like hours and time would pass and I would be like, oh, what just happened? I just went on, just eating dinner and now it’s like nine o’clock. (Non-binary, 19–20)

The time investment of using social media negatively impacted the productivity of the participants and they found it to be stressful. Participants reported the lack of productivity affecting their schoolwork primarily. Some observed a feedback loop in which stress from school would lead to social media use, which ultimately caused a lack of productivity leading to more stress from school.

I think that I’m only on my phone for like 20 minutes . . . like it goes on for like three hours. And then at the end of it. . . I feel like I’m cramming projects. . .on the last second. (Female, 21–22)

Participants attributed their worsening attention spans to their social media use. The immediate gratification associated with using social media, for example, scrolling through Instagram or watching ten 1-min TikTok videos, impacted their ability to focus on important tasks, such as working on assignments or paying attention in class, for long periods of time.

I really don’t have an attention span for anything longer than half an hour, whereas before the pandemic, I felt like I could definitely sit down and study for longer without taking breaks. (Female, 19–20)

### Perceived Advantages of Social Media Use During the Pandemic

#### Social Connectedness

A majority of participants agreed that most communication with family and friends during the pandemic was through social media platforms. With the implementation of public health restrictions, social media became the center of communication by which participants could interact with friends and family who lived abroad. For many participants, social media has become the primary form of communication, replacing more traditional forms such as phone calls or text messages. Participants mentioned the utility of social media for meeting new people, especially in a university setting.

I think without social media it will probably be hard for me to keep in touch with my friends back home and also my family as well. So it’s just like a really convenient thing for me to use. (Female, 19–20)

Some participants acknowledged the positive impact that social media had on their relationships. Social media allowed for easy, reliable, and quick communication with family, friends, and peers which allowed for more frequent contact. New connections were also possible through online chat rooms and group chats.

Social media provided a sense of normalcy that was lost during the pandemic due to public health restrictions. Social media allowed participants to maintain contact with distant family, friends, and peers through posts and messaging, which alleviated symptoms of loneliness and isolation.

I guess, the feeling of wanting to be connected with people. . .if I didn’t have social media, how would I stay close to everybody? And how would everyone know what I’m up to or how would I know what everyone else is up to? Yeah, I think it’s just me wanting to be in the loop, kind of, and being connected with everyone’s lives I guess. (Female, 21–22)

Some participants experienced a sense of community on social media, mainly through group chats with students and friends as well as through interaction with individuals or groups who share similar interests, hobbies, and ideas. However, some participants felt that social media did not provide a sense of community as social comparison and fake portrayals of life are common on the platforms.

I’m watching all these things that I’m interested in and other people are interested in, so it kind of creates this sense of community where. . .if you didn’t already have people in your life. . .who they aren’t interested in the same things as you, you’ll find that community—your sense of community online even if you’re not talking to them one-on-one. (Female, 19–20)

Participants also outlined how social media acts as modern news sources by allowing them to acquire knowledge of events happening domestically and globally. This had implications with respect to the pandemic as social media allowed participants access to information on important topics such as COVID-19-related global and domestic news.

#### Stress Relief

Most participants relieved stress through their social media use despite acknowledging how social media causes stress. Participants reported using social media during stressful moments to distract them from the stressor. Social media use was described as a good mechanism for relaxing and de-stressing, however most participants stated that using social media during stressful moments was not always effective and other methods of stress relief should be pursued.

In a way it allows me to kind of escape my life and see what’s going on in other people’s lives and I’m like, OK, that seems like fun, people are doing great; the pandemic isn’t so bad. So, that’s kind of what social media is for me, it’s kind of a way to escape reality for a few moments, whether that’s studying and me being stressed, I could just be like, OK, time to take a break and just not pay attention to that. (Female, 19–20)

I don’t think it’s the only tool that should be used, I think it’s just one of the tools that is satisfactory to relieving stress, there is so many other things you can do. Even at home, like let’s say cooking, or other hobbies that you can take part of. But social media is just a small, little part of it to help you relieve the stress, I would say. (Female, 17–18)

Some participants gathered inspiration from their social media platforms which benefited them throughout the pandemic. The vast array of content available on social media platforms, including culinary, exercise, educational, and artistic accounts provided participants with inspiration and motivation. One outlined how certain content on social media provided optimism for the future.

it was more of a motivating factor for me because it gave me all these ideas for what I could do once the lockdown restrictions lifted, so I could try to focus on that and be optimistic rather than take that to heart and feel more sad about it. (Female, 19–20)

In addition to inspiration, social media provided participants with a creative outlet where they could post about their hobbies, interests, and ambitions. Some participants used social media to promote their businesses, outlining the benefits of advertising on social media.

I feel like it was a really good creative outlet for a lot of people. A lot of people used it, from what I know, to connect with other people who had their same—like hobbies or interests. . .it was a good platform for like people to like monetize their hobbies, which I know is really big. A lot of people were into like pottery or candle making and they finally took the leap into trying to monetize whatever they were doing. (Non-binary, 19–20)

### Cost–Benefit Analysis

#### Attitudes Toward Increased Usage

Overall, most participants were displeased with their increased social media use during the pandemic lockdowns. Most of the discontent stemmed from an over-reliance on social media, lack of productivity, and mental strain associated with excess use.

sometimes I feel actually really disgusted with myself when I go online for like the amount of time I do and I just feel like I could have been doing something a lot more productive. Even if I wasn’t doing school like I could have done something a lot more. (Male, 17–18)

One participant described a cycle of deleting social media apps only to download them again in the future.

I wake up social media is the first thing that I check on my phone, although I’m not too happy about it. . .Honestly, I’m really not proud of it because I genuinely don’t like social media and if I could I would just delete them and I do have spouts where I do just delete all my social media but then a week will come when there’s like no commitments for me and I’ll just re-download them. (Female, 21–22)

The discussion of whether social media was helpful for participants throughout the COVID-19 pandemic yielded mixed results. Around half of the participants argued that social media was more detrimental than helpful, while the other half argued the opposite. Despite the differences, most participants emphasized the importance of moderation and awareness when using social media.

I can’t say yes and I can’t say no, because, yes, it has helped me, because I’ve been able to communicate with people daily. But no, it hasn’t helped me because I’ve been taking advantage of it and overconsuming it. . .which has had other implications on work and such. (Female, 19–20)

it’s definitely one of those things where you have to balance the good and the bad and I don’t think we would have survived without social media. (Female, 19–20)

## Discussion

This study provided the lived experiences of students attending a large university situated in an urban-rural region approximately 100 kilometers west of Toronto. Through purposive sampling, the study group was diverse and included individuals from various ethnicities, gender identities, sexual identities, and ages. Our study was able to demonstrate how social media can heavily influence the well-being of students in both beneficial and concerning ways.

### Experiences of Loneliness

Loneliness and isolation were experienced by many participants, especially during the initial lockdown phase in Ontario in March 2020. This is consistent with Statistics Canada’s Survey on COVID-19 and Mental Health which found that individuals aged 18 to 24 were most vulnerable to adverse mental health outcomes and experienced loneliness in a higher proportion than other age groups (Statistics Canada, 2021). Participants expressed difficulty in creating new relationships, as well as maintaining already established ones, which may have implicated the onset of loneliness. Young adulthood and adolescence are regarded as developmental periods in which individuals form new relationships (O’Reilly et al., 2018). Individuals in this period of development may be more susceptible to the negative outcomes associated with social media use (Schimmele et al., 2021). As a result of the lack of interaction with others due to social restrictions, as well as the nature of the developmental period, the adverse mental health outcomes reported by Statistics Canada, and described in this study, may be linked to these processes. Our finding is in agreeance with Bonsaksen et al. (2021) whose findings suggest that increased social media use is related to emotional loneliness in young adults. In addition, the awareness that one’s relationships are degrading may exacerbate loneliness and adverse mental health outcomes. Students described how a lack of social interaction in academic situations, including having online classes and the closure of student spaces on campus, hampered their ability to maintain relationships, ultimately degrading them over time. Since social restrictions are beyond the control of the student, their awareness of their degrading relationships may have been more significant. However, participants expressed the utility of social media for communication which prevented symptoms of loneliness. This is contrary to current literature which suggests that decreasing social media use can reduce symptoms of loneliness and depression (Hunt et al., 2018). This contrast could represent the new challenges associated with communication and socialization during the COVID-19 pandemic. In past studies, participants were able to interact with others in-person, with or without social media at their disposal. During the lockdown phases of the pandemic, individuals had few options for communication, and thus social media was utilized, with benefits emerging. The change in conditions is represented in a study conducted by Thygesen et al. (2022) which found that better mental health was related to social media use in the context of maintaining relationships during the pandemic.

### Stress and Social Comparison

The relationship between social media and stress is dynamic as social media helps reduce stress in certain situations while inducing stress in others. Social comparison led to stress and was prevalent in the interviews and impactful enough to justify its own theme. The experiences of the participants demonstrate that social comparison through social media can have adverse mental health effects. Bonsaksen et al. (2021) found that young adults’ inclination to social comparison may explain increased loneliness among that age group. Our findings support this hypothesis as most participants reported experiencing loneliness and isolation during the pandemic while also citing the negative impacts of social comparison through social media. However, in contrast to the literature, the participants in the current study expressed comparisons based on lifestyle more frequently than comparisons of body image, suggesting that the pandemic influenced how individuals compare themselves to others online. For example, due to the severe public health restrictions in Ontario, participants frequently reported feeling lonely or distraught after viewing individuals on social media partaking in events such as traveling or large social gatherings. Although this likely occurred prior to the pandemic, social restrictions may have exacerbated these experiences of comparison. Social media allowed individuals to observe the lives of others, specifically those who were living in restriction-free or fortunate circumstances. Participants compared themselves to others who they perceived as being in a more advantageous position than themselves as these individuals were able to portray their restriction-free life on social media. This supports the upward social comparison theory described in the literature, which posits the effects of screen time on mental health are a product of the content consumed by the user, ultimately contributing to adverse mental health outcomes such as depression (Boers et al., 2019). Contrary to existing studies (Chew et al., 2020; Savitsky et al., 2020; Wang et al., 2022), our findings show that the COVID-19 pandemic did not act as a stressor past the initial phases and lockdown periods of the pandemic. The consequences of the outbreak provided more stress to students than the actual pandemic itself. This is in agreeance with Visser and Law-van Wyk (2021) who found that mental and emotional health was not impacted by potential infection.

### Impact on Academic Performance

The literature regarding the relationship between social media and academic performance suggests that social media has negative implications on academic outcomes for adolescents and/or university students (Kolhar et al., 2021; Tsitsika et al., 2014). In accordance with the literature, participants in our study were concerned their social media use adversely affected their academic performance and school-related stress. One study found that heavy social media use was associated with internalizing problems and poor academic performance in adolescents (Tsitsika et al., 2014). Internalizing problems includes adverse mental health conditions such as anxiety and depression, which have negative implications for well-being (Luijten et al., 2021). In addition to anxiety and depression, students are also at risk of experiencing stress due to both academic and non-academic reasons, such as course workload, parental expectations, competitions, and more (Ramón-Arbués et al., 2020). Participants in our study expressed how both social media and the COVID-19 pandemic exacerbated the stress experienced through their academics. In agreeance with the literature (Visser & Law-van Wyk, 2021), many participants in our study explained how social media distracted them from completing assignments or studying. This could be due to how the pandemic altered the learning environment of students rapidly and resulted in online learning and communication. Many participants experienced burn-out where the constant use of technology for educational, social, and communicative purposes resulted in difficulties in focusing. This is consistent with Visser and Law-van Wyk (2021), who found that the pandemic was associated with reduced academic ability through difficulties in self-studying and online learning. Social media impacts this process as students resort to using social media as a form of stress relief and distraction when their academics act as a stressor, despite the disadvantages described prior. Our findings are in agreeance with Nesi (2020) who posits that social media can provide benefits including entertainment, creative expression, and social connection. In our study, benefits such as creative expression, entertainment, inspiration, and social connectedness were reported and ultimately provided stress relief.

### Self-Perceived Negative Habit Development

Most participants were unhappy with their time spent on social media platforms. Consistent with Visser and Law-van Wyk (2021) many participants outlined challenges that could have adverse effects on their academics, such as a worsening attention span and reduced productivity. Interestingly, most participants expressed experiencing increased stress from their academics as a result of the negative habits developed from using social media. This is reflective of the displacement theory which suggests that all screen time results in negative mental health outcomes as it displaces beneficial activities that promote well-being, including physical exercise or engaging in meaningful work (Boers et al., 2019). Furthermore, remote course delivery required students to be online for a significant amount of the day. The findings of Lee et al. (2022) state that the quick and unexpected transition to online learning and communicating may have triggered mental health issues. In our study, most participants reported feeling fatigued by their online academic environment, resulting in social media use as a distraction and reduced productivity after class due to burn-out. A dangerous cycle can be seen in this case as students revert to social media due to academic stress, which causes more academic stress due to poor time management and so on. Our results indicate an interplay between social media, COVID-19, and academics, and the resulting outcomes of stress and internalizing problems.

### Social Connectedness

Participants described many advantages associated with social media use including communication and forging a sense of community. This is in accordance with the literature suggesting positive potential for relationship building on social media, providing users with feelings of belonging and acceptance (Clark et al., 2018; Nesi, 2020). In addition, most participants expressed the utility of social media for maintaining relationships and staying connected with friends, family, and peers during the lockdown periods of the pandemic. This is a representation of active social media use as communication requires engagement from both parties as described by Thygesen et al. (2022). Compared to passive social media use, active social media use during the pandemic may have stimulated individuals socially acting as a potential protective factor against loneliness. Existing studies have found that social media acts as a mechanism of social connection, thereby possibly reducing symptoms of loneliness (Bonsaksen et al., 2021; Maclean et al., 2022; Sarangi et al., 2022). Furthermore, our findings are consistent with those of Lee et al. (2022) showing that although social media has benefits related to social connectedness, excessive use of the platforms can be detrimental.

### Importance of Moderation and Consumed Content

Participants emphasized the importance of balance and moderation when using social media as they experienced both the benefits and harms of its use. Since our study took place in February 2022, the participants were able to consider multiple perspectives throughout the pandemic including the early phases as well as the less restrictive phases. Therefore, the theme of moderation was likely developed over a considerable time span which underlies its importance. In addition, the importance of moderation can be applied to the type of content consumed on social media. For example, social comparison may be more likely to occur when the individual is consuming content related to unrealistic lifestyles. However, some participants in our study identified social media as useful for expressing their interests and hobbies and advertising small businesses. The literature also suggests that social media can be used as a creative outlet (Nesi, 2020). Since public health restrictions prevented social gatherings, social media became the environment for creativity for many. Subsequently, many participants reported using social media to relieve stress, and the expression of creativity online may have been one mechanism through which this occurred.

### Social Media, Stress, and Social Justice

The death of George Floyd in 2020 was particularly distressing for one racialized participant in our study. Floyd’s encounter with the police and the resulting social tension and discussion could be found on social media, specifically Twitter (Thelwall & Thelwall, 2021). Our study demonstrates how racialized individuals may be subjected to unique stressors when using social media not only during times of social unrest but also in general as outlined by Matamoros-Fernández and Farkas (2021). Interestingly, according to Thelwall and Thelwall (2021), the death of George Floyd triggered an increase in Tweets mentioning both COVID-19 and racism. Many of which outlined the disproportionate impact of COVID-19 on the African American community. In this sense, the interplay between social media and social tension resulted in forms of activism. Thelwall and Thelwall (2021) state that the death of George Floyd amplified the anti-racism discussion on social media.

### Policy Implications

Currently, digital and media literacy programs are present in primary and secondary school curricula across Canadian provinces. In Ontario, the topics of digital media and body image are introduced to students in elementary school (Media Smarts, n.d.). Our findings suggest education pertaining to social and digital media should be enhanced and cover a broader scope of topics. For example, many participants in the current study expressed how social media education revolves around body image but lacks in educating on overconsumption, and adverse mental health outcomes. Although the participants in our study were university students, their exposure to social media likely occurred before attending a university suggesting that comprehensive social media-related education should begin in primary and secondary school. It is crucially important for university administrators to understand how they can support students who are struggling with mental health during the pandemic. Participants in our study did not identify, nor mention, Wilfrid Laurier University’s mental health support systems which may be indicative of an underutilization of the resources. Our sample size is small, and no direct conclusions can be found from this observation. The American College Health Association (2019) found within Canadian reference data that approximately 45% of students had low to very low awareness of how to access mental health services or resources on campus. In the same study, about 19% of students disagreed that their university environment was one where their mental health was supported; 6.5% strongly disagreed. University administrations must not only provide adequate resources but also promote the use of these resources so that unfamiliar students can be aware of them. Our study found that social media use among university students has drastically increased since the start of the pandemic which suggests that social media can be used as a potential resource for mental health support. According to Nowrouzi-kia et al. (2021), ICT, including mental health support apps, can provide solutions that university administrations cannot. Although it is somewhat counterproductive considering social media use is linked with adverse mental health outcomes, students already use social media extensively. Thus, ICT should be considered as a support system and universities should seek to develop applications, or social media pages, that target the mental health of students.

As its own entity, social media can impact the mental health of individuals in various ways. However, the COVID-19 pandemic changed the behaviors of many with respect to social media, thus altering the complexity of the relationship between social media and well-being. Many participants illustrated that their mental health was both damaged and aided by social media during the pandemic. However, they agreed that the interplay between social media and the pandemic influenced their mental health. Therefore, healthcare providers and policy makers should provide more focus on mental healthcare with regard to access and education. During the COVID-19 pandemic, physical health was of primary concern as social restrictions were implemented to prevent the spread of the virus. Our findings indicate that there should be a strong focus on mental health and how digital and social media can impact it.

## Study Limitations

This study is not representative of the Canadian university student population and is not generalizable across larger populations nor is it meant to be. It was designed to provide an in-depth analysis of the experiences of these 15 participants and is bound to have some applicability in other similar settings. Despite the diversity in the sample, a majority of participants were female. The results demonstrate how the impact of social media on students is multifactorial and thus each student, and user of social media, will be influenced differently. Therefore, documenting more experiences representing greater population diversity would further develop the literature and provide a more holistic understanding of the situation. Hosting interviews through a secured online platform posed limitations as well. Although participants were given the option to activate their cameras, many opted not to. Thus, interpreting body language was impossible for the interviewer, and determining the level of engagement and focus of the participants was solely achieved through verbal cues. Furthermore, this study only included individuals who noticed an increase in their social media usage. Thus, this study did not consider the perceived effects of decreased social media usage during the pandemic. A separate study should be conducted to evaluate these perceived effects allowing for a comprehensive body of work on this topic.

## Conclusion

For this group of university students in Ontario Canada, different aspects of social media were either advantageous or disadvantageous throughout the pandemic. Social media use for communication and stress relief were identified as the major advantages, whereas social comparison, negative habit development, and impact on the ability to focus were identified as disadvantages. Participants considered social media use as a cost-benefit analysis with some believing the benefits to outweigh the concerns, and others believing the opposite. These findings indicate that education regarding social media usage should move beyond social comparison with respect to body image and should encompass a broader scope of topics including overconsumption and adverse mental health outcomes while also exploring how the benefits of social media can be acquired through appropriate use. Future research should seek to understand how social media impacts the attention spans and productive behaviors of students as the long-term impacts of the pandemic are yet to be seen.

## Appendix

### Interview Guide

1. Tell me about a typical day for you and how social media plays a role in your day?
  What apps/platforms do you use most frequently?
  Do these platforms serve different purposes?
   If so, please explain what platforms you use and for what reason.
   Please rank which apps you used the most throughout the pandemic? Number one being the most used platform.
2. What were some of the reasons for your social media usage?
  What draws you into using social media?
  Do you find it particularly useful for communicating with others?
  Do you use social media as a distraction from reality?
  Does social media provide you with a sense of community?
3. How has your social media consumption changed since the beginning of the pandemic?
  Would you say it has increased, decreased, or stayed the same?
  If your social media consumption has changed, why do you think that is?
  How do you feel about the change? probe for concerned, pleased, or indifferent
4. During the initial and subsequent lockdowns, how did you communicate with your friends/family/peers during the lockdowns?
  How did your relationships change during the lockdowns? Were they more difficult to maintain?
  How would you describe any symptoms of isolation and/or loneliness? Please explain how social media contributed to these symptoms or possibly helped relieve them.
5. Do you think people use social media to deal with stress?
  How about you? How does stress figure into your social media use?
  Possibly stress from school or Covid-19-related issues?
  Do you think social media has caused you stress?
   If so, in what ways has social media caused you stress?
  Do you think social media has caused or relieved more stress?
   Is it an effective tool for dealing with stress?
6. What would you say were some of the benefits of using social media during the pandemic?
  Were these benefits unexpected?
  Did you notice these benefits having a positive effect on your life?
  Do you think these benefits will continue to have a positive impact in a post-pandemic world?
7. What would you say were some of the concerns or disadvantages of using social media during the pandemic?
  Were these negative effects expected?
  How would you describe these negative impacts on your life?
  Do you think these harms will have a long-term effect on you?
8. Overall, do you think social media has helped you throughout the pandemic?
  Would you consider it an effective tool in coping with a decreased social life? Why or why not?
9. Do you think we need to talk more about mental health and social media usage, and how it relates to the pandemic? If so, what are the key messages that need to be said?
